# Prospective Randomized Control Trial Comparing Effect of Dexamethasone Versus Fentanyl as Adjuvants to Intrathecal Bupivacaine for Orthopedic Surgery

**DOI:** 10.7759/cureus.13949

**Published:** 2021-03-17

**Authors:** Harjot Kaur, Rajesh Misra, Shelly Mittal, Gur Aziz Singh Sidhu

**Affiliations:** 1 Anesthesia, Artemis Hospitals, Gurgaon, IND; 2 Orthopedics, Medanta Hospital, Gurgaon, IND

**Keywords:** spinal anesthesia, adjuvants, dexamethasone, fentanyl, motor blockade, sensory blockade

## Abstract

Introduction

Spinal anesthesia is the most consistent block for lower limb orthopedic surgeries. We conducted this randomized prospective study to evaluate comparative efficacy of intrathecal dexamethasone with fentanyl and normal saline as adjuvants to hyperbaric bupivacaine in spinal anesthesia administered to patients scheduled for lower limb orthopedic surgery.

Materials and methods

105 patients scheduled for lower limb orthopedic surgeries under spinal anesthesia were included in this clinical trial. After randomization, patients received an intrathecal injection of hyperbaric bupivacaine (12.5 mg) with 4 mg of dexamethasone in group I, hyperbaric bupivacaine (12.5 mg) with 25 ug fentanyl with 0.5 ml of normal saline in group II and hyperbaric bupivacaine (12.5 mg) with normal saline (1 ml) in group III, so as to make volume of drug equal in all three groups. The observer evaluated the sensory and motor blocks and other parameters like time to self-void, stay in post-anesthesia care unit (PACU) and complications.

Results

The total duration of sensory blockade was found to be 311.43, 197.86 and 115.29 minutes and motor blockade of 223.43, 163.86 and 83.0 minutes in groups I, II and III respectively. The PACU stay was 233.14, 173.86 and 93.00 minutes in groups I, II and III, respectively. The average time to self-void was 400.00, 315.29 and 203.00 in three groups, respectively.

Conclusion

Intrathecal dexamethasone seemed to be an effective adjuvant to spinal bupivacaine as it prolongs the duration of analgesia, stable hemodynamic profile with minimal side effects. Further studies are required to evaluate the optimum dose and long-term safety of intrathecal dexamethasone.

## Introduction

Spinal anesthesia is the most consistent block for lower limb orthopedic surgeries as it provides excellent anesthesia, minimal cognitive dysfunction, and post-operative analgesia. Moreover, less incidence of post-operative nausea and vomiting, cardiovascular or respiratory complications and better post-operative pain relief and the patient's ability to communicate are added advantages [1]. Various agents have been used in subarachnoid block as adjuvants such as epinephrine, phenylephrine, clonidine, opioids, etc. to prolong the effect of spinal anesthesia [1,2]. However, every adjuvant has its own advantages and disadvantages. The use of epinephrine(> 10 microg/mL) as adjuvant causes tachycardia, pallor, and hypertension; which can be detrimental in patients with cardiovascular disease [3]. Intrathecal opioids cause pruritus, urinary retention, respiratory depression and occasionally severe nausea and vomiting that may limit their use [4].

Recently, literature reports few studies on prolongation of sensory blockade in peripheral nerves both in quantitative as well as qualitative way after addition of corticosteroids [1,5]. Local anesthetics act by blocking the sodium channels, whereas dexamethasone relieves pain by reducing inflammation and blocking transmission of nociceptive C-fibers and by suppressing ectopic neural discharge in abnormal nerves [6]. It has been shown that the duration of post-operative analgesia was prolonged when dexamethasone was given as an adjunct for peripheral nerve blocks [7]. We found that a very few studies in the literature reported the intrathecal use of dexamethasone as an adjuvant [8,9]. Hence keeping this in view, we conducted this study to evaluate comparative efficacy of intrathecal dexamethasone, fentanyl and normal saline as adjuvants to hyperbaric bupivacaine in spinal anesthesia administered to patients scheduled for lower limb orthopedic surgery.

## Materials and methods

After the approval from the scientific and research ethics committee, written informed consent was obtained from each patient enrolled in the study. Patients with American Society of Anesthesiologist (ASA) class I-II, between 18 and 65 years old, scheduled for lower limb orthopedic surgeries under spinal anesthesia were included in this clinical trial. Patients with contraindications to spinal anesthesia, neurologic disease (multiple sclerosis, symptomatic lumbar herniated disc, spinal stenosis), ﬂuid restriction (cardiac or renal insufficiency), allergy or intolerance to local anesthetics, history of long term steroid therapy, opium addiction or refusal to participate in the study were excluded from study.

Premedication with parenteral ranitidine 50 mg and parenteral ondansetron 4 mg was given in pre-operative room. Standard monitoring was used throughout the procedure, including non-invasive arterial blood pressure, electrocardiogram (five leads), and pulse oximetry. Sedation was provided at the discretion of the anesthesiologist (midazolam 0.025 to 0.05 mg/kg IV before or immediately after the spinal).

Spinal anesthesia was performed under sterile conditions after local inﬁltration of the skin with 2% lidocaine. With the patient in the sitting position, the subarachnoid space was entered at the L2-3, L3-4, or L4-5 interspace via the midline approach using a 27G Quincke spinal needle. We used computer randomization in which the patients and observer were blinded (double-blinded study). According to randomization, patients received an intrathecal injection of hyperbaric bupivacaine (12.5 mg) with 4 mg of dexamethasone in group I, hyperbaric bupivacaine(12.5 mg) with 25 ug fentanyl with 0.5 ml of normal saline in group II and hyperbaric bupivacaine (12.5 mg) with normal saline (1 ml) in group III, so as to make volume of drug equal in all three groups. After the completion of the spinal injection, the patients were immediately placed supine. The observer (who was blinded and was not involved in the procedure) evaluated the sensory and motor blocks every one minute for 5 minutes or till the desired level of block was achieved, then every 15 minutes during the surgery and postoperatively until the sensory block had regressed to the L1 dermatome. The sensory level of the block was assessed in a caudal to cephalad direction using the loss of sensation to pinprick, and the forearm was used as the reference point. The motor block was assessed using the Modiﬁed Bromage scale. Readiness for surgery was deﬁned as loss of sensation to pinprick at T10. During surgery, evaluation of the motor block was suspended until the end of the procedure.

If additional sedation was needed, midazolam 0.025 to 0.05 mg/kg IV or propofol 25 to 50 mcg/kg/min IV was administered. The total dose of any given medication was recorded. If the patient still felt pain, general anesthesia was provided and the patient was excluded from the study.

The occurrence of clinically relevant hypotension (deﬁned as a decrease in systolic arterial blood pressure 20% from baseline values) was treated with IV injection mephentermine 6 mg. Clinically relevant bradycardia (deﬁned as heart rate <50 beats/min) was treated with injection atropine 0.6 mg IV. The cumulative dose of any of these medications or sedatives was recorded. The duration of sensory and motor block was recorded along with the side effects or complications (nausea, vomiting, hypotension, bradycardia, etc.) were recorded along with cumulative dose of additional agents used. The duration of sensory block was defined from peak of sensory block up to regression to L1 level or when the patient’s felt pain in the field of surgery. Motor block duration was defined from peak of motor block (Modified Bromage score 3) to complete motor recovery (Modified Bromage score 0).

Patients were discharged from the post-anesthesia care unit (PACU) once they attained all of the following criteria: stable vital signs, signs of regression of the motor block (Bromage 0 to 2), no analgesia needed within the previous 20 min and normal consciousness. In the post-operative period, time to self-voiding of urine was noted. Incidence of post-operative shivering, nausea, vomiting, hypotension and bradycardia was recorded and compared. In post-operative period patients were assessed for pain by visual analogue scale (VAS), rescue analgesia and time to first post-operative analgesia were recorded. In post-operative period patients were assessed for pain by visual analogue scale (VAS) one hourly for the first four hours and then at four hourly intervals till 12hours or till VAS more than 4. Rescue analgesia was given in form of injection tramadol (100 mg in 100 ml saline) intravenously if pain on VAS was more than 4.

The continuous data were shown as mean with standard deviation and categorical data was represented as absolute numbers and percentages. For continuous data, the Kolmogorov-Smirnov tests were performed to assess normality and where appropriate the data was analyzed with required statistical tests and descriptive statistics. Parametric data were analyzed with analysis of variance (ANOVA). Non-parametric data were analyzed Kruskal Wallis test. Nominal categorical data between the groups were compared using Chi-square test or Fisher’s exact test as appropriate and use the correlation coefficient to observe the linear relationship. 

## Results

The patients in all the three groups were comparable with respect to age, sex, ASA class and the length of surgical procedures. The onset of sensory and motor block was comparable among all the groups. The mean time of onset of sensory block (TOSB) in group I, group II and group III were 6.85 (±1.21), 7.65 (±1.25) and 7.20 (±1.47) minutes respectively. The mean time of onset of motor block (TOMB) in groups I, II and III was 9.22 (±1.47), 9.82 (±1.27) and 8.94 (±1.34) minutes, respectively. However, no statistically significant difference was found in three groups with relation to TOSB and TOMB. The duration of surgery in three the groups was found to be comparable with 86.14 (±15.02) minutes in group I, 88.29 (±13.39) minutes in group II and in group III the mean surgical duration was 86.20±13.45 minutes which was statistically insignificant (p = 0.211).

**Figure 1:**
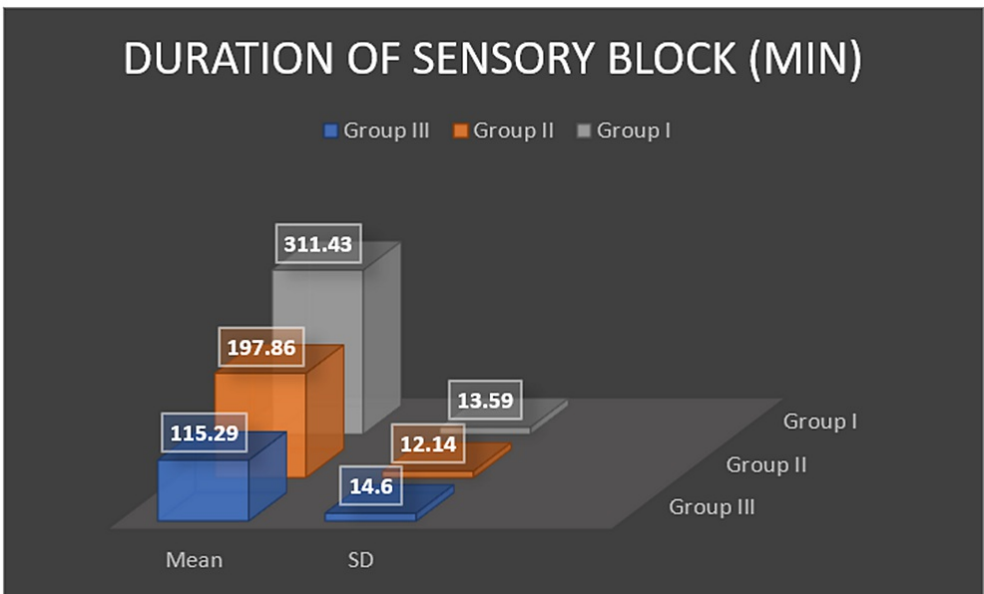
Duration of sensory block.

**Figure 2:**
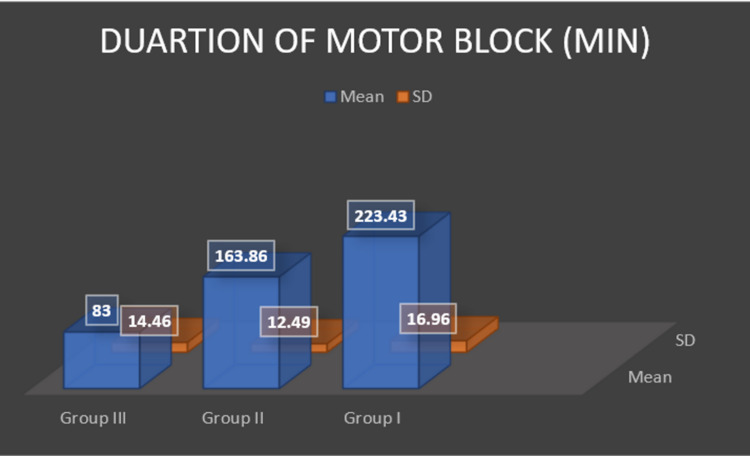
Duration of motor block.

The total duration of sensory blockade was found to be 311.43 (SD 13.59) minutes in group I, 197.86 (SD 12.14) minutes in group II and the group III reported 115.29 (SD 14.60) minutes of sensory blockade and was statistically significant (p < 0.0001) (Figure 1).The total duration of motor blockade was found to be 223.43±16.96 minutes in group I, 163.86±12.49 minutes in group II and the group III reported 83.0±14.46 minutes of motor blockade and the difference was statistically significant (p < 0.0001) (Figure 2).

The patients in group III stayed in the PACU for 93.00 (±14.46) minutes on the contrary the length of PACU stay was 173.86 (±12.49) minutes in the patients belonging to group II and 233.14 (±17.28) minutes in group I with p < 0.0001. The time taken to self-void was 400.00 (±29.13) minutes in group I, 315.29 (±21.18) minutes in group II and group III patients took 203.00 (±19.71) minutes to self-void. The difference amongst different groups was statistically significant (p < 0.0001).

**Figure 3:**
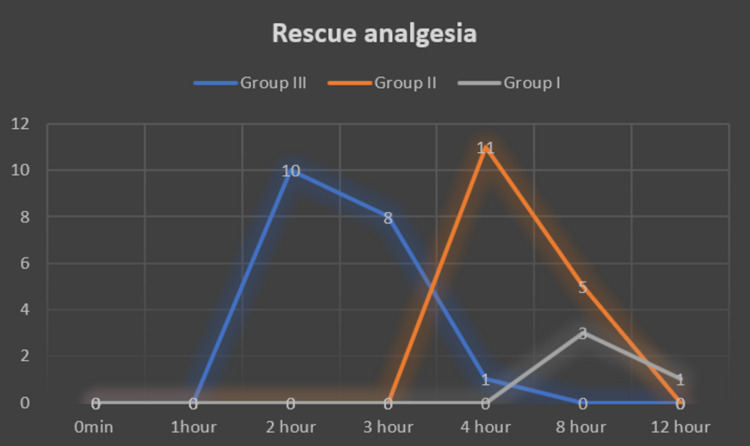
Rescue analgesia.

Thirty (28.57%) patients required rescue analgesia within four hours after the surgery. Rescue analgesia was given within two hours in 10 patients, eight patients had pain within three hours after surgery and only one patient did not have pain until four hours after surgery in group III. 11 patients in group II required rescue analgesia within four hours; however, in group I, none required rescue analgesia in first four hours (Figure 3). Prolonged duration of analgesia with significantly delayed time of administration of rescue analgesia was observed in group I compared to group II and III. The difference in time of first rescue analgesia in groups I, II and III was statistically significant (p < 0.0001).

**Figure 4:**
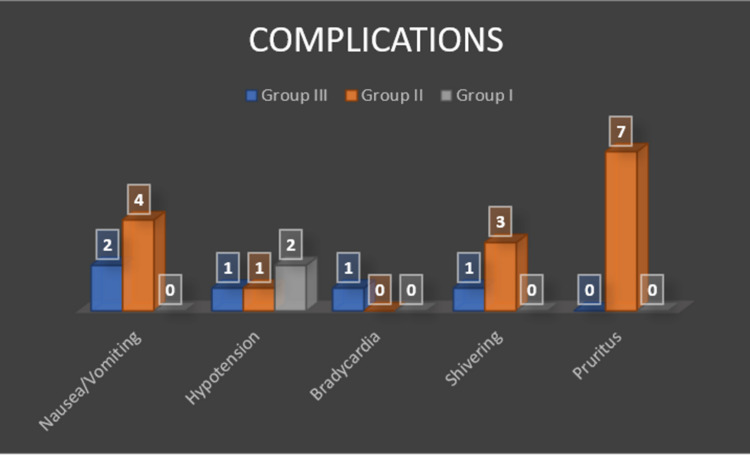
Post-operative complications.

In group III, two patients had nausea/vomiting, one patient each had bradycardia, hypotension and shivering. In group II, seven patients had pruritus, four had nausea/ vomiting, three patients had shivering and only one patient had hypotension. However, in group I only two patients had hypotension and no patients had complaints of nausea/vomiting, bradycardia, shivering or pruritus (Figure 4).

## Discussion

Early and effective management of postoperative pain allows early mobilization of patient after orthopedics surgery thus preventing the associated co-morbidities. Different combinations of drugs are used to target different phases of pain pathway from perception to central modulation. The addition of intrathecal adjuvants in spinal anesthesia offers hemodynamic stability along with prolongation of sensory block, longer postoperative analgesia and fewer undesirable effects [10].

Literature reported various studies showing the analgesic effects of steroids in neuraxial and peripheral blocks [11,12]. The results of our study demonstrated that supplementation of spinal bupivacaine with dexamethasone significantly prolonged sensory block, and postoperative analgesia compared with intrathecal bupivacaine alone or when fentanyl was used as an adjuvant with intrathecal bupivacaine, without any effect on the onset time of sensory or motor block in lower limb orthopedic surgeries.

A study by Fayyaz et al. compared the mean duration of analgesia with intrathecal bupivacaine alone versus intrathecal bupivacaine plus dexamethasone, for elective caesarean section [13]. They concluded that dexamethasone and hyperbaric bupivacaine provided 391±25.51 minutes of analgesia as compared to hyperbaric bupivacaine alone 179.43±23.32 minutes which was statistically significant. Our study reported similar results as total duration of sensory blockade was found to be 115.29±14.60 minutes in control group, 197.86±12.14 minutes in fentanyl group and the dexamethasone group reported 311.43±13.59minutes of sensory blockade which was statistically significant (p < 0.0001). Our cohort comprised of 105 patients divided in three groups, whereas Fayyaz and colleagues had 60 patients divided into two groups.

In another randomized, prospective, double-blinded clinical trial, Movafegh and coworkers evaluated the prolongation of lidocaine spinal anesthesia by intrathecal administration of dexamethasone in 90 patients scheduled for orthopedic surgery [14]. They reported that the duration of sensory block was significantly longer in the lidocaine-epinephrine and lidocaine-dexamethasone groups than the lidocaine alone group. These results were similar to our study with respect to motor and sensory blockade; control group: 83.0±14.46 minutes vs 115.29±14.60 minutes; fentanyl group: 163.86±12.49 minutes vs 197.86±12.14minutes and the dexamethasone group: 223.43±16.96 vs 311.43±13.59 minutes. Thus, contributed to our hypothesis that the addition of dexamethasone intrathecally as an adjuvant to bupivacaine in spinal anesthesia prolongs the duration of sensory and motor blockade. The only difference in above studies was, we used hyperbaric bupivacaine whereas Movafegh and colleagues used lidocaine for spinal anesthesia.

Kikuchi A et al. studied 25 patients with post-herpetic neuralgia (PHN) of more than 1 year who were randomly allocated to one of two groups: an intrathecal group (n = 13) and an epidural group (n = 12). The improvements were much greater in the intrathecal group than in the epidural group at all-time points after the end of treatment (p < 0.005) [15]. Moreover, IL-8 in the CSF decreased significantly in the intrathecal group as compared to the epidural group at the l-week time point (p < 0.01), whereas the other cytokines were undetectable suggesting that intrathecal MPA improves analgesia by decreasing an ongoing inflammatory reaction in the CSF [15]. This indirectly supported our study as the time to rescue analgesia was much higher in dexamethasone group as compared to fentanyl and control groups. Also, intrathecal dexamethasone may influence intraspinal prostaglandin production. Corticosteroids are capable of reducing prostaglandin synthesis by inhibition of phospholipase A2 through the production of calcium-dependent phospholipid-binding proteins called annexins and by the inhibition of cyclooxygenases during inflammation [16]. 

Numerous other studies in literature can be found supporting that steroids have powerful anti-inflammatory as well as analgesic property [17,18]. Mirzaie et al. reported that corticosteroids and bupivacaine can diminish the incidence of back pain after laminectomy in the immediate postoperative period [19]. Kotani et al. administered methylprednisolone with bupivacaine intrathecally in patients with PHN. They concluded that this combination induced excellent and long-lasting analgesia [20].

In a randomized study by Thomas and Beevi, 94 patients undergoing laparoscopic cholecystectomy were divided into three groups and post-operative morphine requirements, VAS pain scores at rest and with effort, and time to first analgesic administration were recorded [21]. They reported total morphine consumption for the first 24 hours following surgery was lower in both epidural dexamethasone groups (D1, D2) compared to the control group (p < 0.05). The percentage reduction in morphine consumption in Group D1 was 53.9% and in Group D2 was 52.9% in the first 24 hours. These values were in accordance to our observations. About 30 (28.57%) patients in our study required rescue analgesia within four hours after the surgery. Rescue analgesia was given within two hours in 10 patients, eight patients had pain within three hours after surgery and only one patient did not have pain until four hours after surgery in control group. Eleven patients in fentanyl group required rescue analgesia within four hours; however, in dexamethasone group, none required rescue analgesia in the first four hours. Prolonged duration of analgesia with significantly delayed time of administration of rescue analgesia was observed in dexamethasone group compared to fentanyl and normal saline groups.

Similarly, Bani-Hashem et al. conducted a randomized prospective clinical trial on 50 patients scheduled for orthopedic surgery under spinal anesthesia [9]. Sensory block duration in the case group was 119±10.69 minutes and in the control group was 89.44±8.37 minutes which was significantly higher in the case group (p < 0.001). Although our results were equivocal on sensory block duration of 115.29±14.60 minutes in control group, 197.86±12.14 minutes in fentanyl group and 311.43±13.59 minutes in dexamethasone group, they reported the frequency of complications was not different between two groups but our cohort showed statistically significant observations with respect to complications like nausea/ vomiting, bradycardia, shivering, etc. In our study, two patients in control group, four patients in fentanyl group complained of nausea/ vomiting; pruritus was reported in seven patients in fentanyl group; shivering was observed in three patients in fentanyl group and one patient in control group; one patient in control group had bradycardia which was managed with injection atropine 0.6 mg; two patients in dexamethasone group had hypotension whereas one patient each in fentanyl and control group had hypotension which was managed with injection mephentermine 6 mg. None of the patients in the dexamethasone group had nausea or vomiting. This result may be due to the antiemetic effect of dexamethasone, which has been demonstrated in other studies using perioperative corticosteroids [22,23].

In a double-blind, placebo-controlled study by Bisgaard and colleagues, 88 patients were randomized to intravenous dexamethasone (8 mg) or placebo 90 minutes before lap cholecystectomy and the duration of stay at the PACU in dexamethasone group was 75 minutes (range 25-169) and in placebo group was 70 minutes (range 15-240 minutes) which was not significantly different between the two groups [24]. On contrary, in our study dexamethasone group stayed on an average for 233.14±17.28 minutes, fentanyl group for 173.86±12.49 minutes and 93.00±14.46 minutes in control group in PACU and this difference was significant statistically. This difference was mainly due to our strict discharge criteria i.e stable vital signs, signs of regression of the motor block (Bromage 0 to 2), no analgesia needed within the previous 20 min, and once patients attained all of the following criteria then only, they were shifted. Also, all patients voided normally without any need for catheterization. The control group took 203.00±19.71 minutes, the fentanyl group took 315.29±21.18 minutes at self-voiding of urine. However, patients in dexamethasone group, took 400.00±29.13 minutes to self-void and the difference was statistically significant in our cohort (p < 0.0001). The reason for a longer time to self-void may be due to prolonged time taken for regression of sensory and motor blocks in dexamethasone group.

Literature reported contrary studies regarding the safety profile of intrathecal steroids. In animal experiments, triamcinolone did not induce spinal neurotoxicity, whereas repeated high-dose intrathecal injections of betamethasone caused histopathological changes of the spinal cord [25,26]. However, intrathecal injection of steroids was frequently used for the treatment of mumps meningitis, chronic lymphocytic leukemia and central nervous involvement in lupus erythematosus [27]. We did not report any considerable complication for intrathecal dexamethasone. Similarly, Kotani et al. found no complication in patients who received intrathecal methyl prednisolone [20]. Also, Sugita and colleagues did not find any complication after intrathecal injections of dexamethasone in patients with posttraumatic visual disturbance [28]. However, further studies with longer follow-up are needed to verify the safety profile of intrathecal dexamethasone.

## Conclusions

Although both fentanyl and dexamethasone prolong the duration of sensory and motor blockade in spinal anesthesia; however, dexamethasone could be a better alternative as it provides stable hemodynamic profile with minimal side effects. Longer motor blockade, time to self-void and longer PACU stay might limit its use unless a better dose can be found. So, further studies are needed to evaluate the optimal dose as well as long-term safety of intrathecal dexamethasone.
